# Transcriptome-wide RNA processing kinetics revealed using extremely short 4tU labeling

**DOI:** 10.1186/s13059-015-0848-1

**Published:** 2015-12-17

**Authors:** J. David Barrass, Jane E. A. Reid, Yuanhua Huang, Ralph D. Hector, Guido Sanguinetti, Jean D. Beggs, Sander Granneman

**Affiliations:** Wellcome Trust Centre for Cell Biology, University of Edinburgh, Edinburgh, EH9 3BF UK; School of Informatics, University of Edinburgh, Edinburgh, EH8 9AB UK; Centre for Synthetic and Systems Biology (SynthSys), University of Edinburgh, Edinburgh, EH9 3BF UK; Present Address: Institute of Neuroscience and Psychology, University of Glasgow, Glasgow, G12 8QB UK

**Keywords:** Cryptic transcripts, Metabolic labeling, RNA sequencing, Splicing, Yeast

## Abstract

**Background:**

RNA levels detected at steady state are the consequence of multiple dynamic processes within the cell. In addition to synthesis and decay, transcripts undergo processing. Metabolic tagging with a nucleotide analog is one way of determining the relative contributions of synthesis, decay and conversion processes globally.

**Results:**

By improving 4-thiouracil labeling of RNA in *Saccharomyces cerevisiae* we were able to isolate RNA produced during as little as 1 minute, allowing the detection of nascent pervasive transcription. Nascent RNA labeled for 1.5, 2.5 or 5 minutes was isolated and analyzed by reverse transcriptase-quantitative polymerase chain reaction and RNA sequencing. High kinetic resolution enabled detection and analysis of short-lived non-coding RNAs as well as intron-containing pre-mRNAs in wild-type yeast. From these data we measured the relative stability of pre-mRNA species with different high turnover rates and investigated potential correlations with sequence features.

**Conclusions:**

Our analysis of non-coding RNAs reveals a highly significant association between non-coding RNA stability, transcript length and predicted secondary structure. Our quantitative analysis of the kinetics of pre-mRNA splicing in yeast reveals that ribosomal protein transcripts are more efficiently spliced if they contain intron secondary structures that are predicted to be less stable. These data, in combination with previous results, indicate that there is an optimal range of stability of intron secondary structures that allows for rapid splicing.

**Electronic supplementary material:**

The online version of this article (doi:10.1186/s13059-015-0848-1) contains supplementary material, which is available to authorized users.

## Background

The RNA levels detected in cells at steady state are the consequence of multiple dynamic processes within the cell. Eukaryotic genomes are pervasively transcribed, but the accumulation of many transcripts is limited by processes that regulate their synthesis and decay [1]. In addition, most primary transcripts undergo processing events. For example, small nuclear RNAs (snRNAs), small nucleolar RNAs (snoRNAs), transfer RNAs (tRNAs), microRNAs (miRNAs), small interfering RNAs (siRNAs) and some long non-coding RNAs (lncRNAs) are often produced by the post-transcriptional processing of short-lived longer transcripts that are more readily detected in the absence of degradation or processing factors [2]. A number of different classes of lncRNAs have been described in eukaryotes [3, 4]. The best characterized examples in yeast are the stable unannotated transcripts (SUTs), cryptic unstable transcripts (CUTs) and Xrn1-sensitive unstable transcripts (XUTs), three types of lncRNAs thought to have different stabilities [4]. SUTs are readily detectable in wild-type cells and share many similarities with messenger RNAs (mRNAs), whereas CUTs are generally more unstable and frequently only detectable in cells lacking the nuclear exosome components. XUTs are likely (primarily) degraded in the cytoplasm because they accumulate in the absence of the cytoplasmic exoribonuclease Xrn1 [5].

In the case of intron-containing genes, the level of mature transcripts is influenced by splicing, as well as by synthesis and decay. Splicing of pre-mRNAs (precursors of messenger RNAs) [6] occurs in the nucleus, often co-transcriptionally [7]. The spliced mRNA is exported to the cytoplasm where it can be translated, whereas the excised intron, which has a branched, lariat structure, is rapidly debranched and degraded. Measurements of in vivo RNA processing rates and efficiencies depend on the ability to estimate the levels of the unprocessed precursors and processing intermediates in cell extracts; however, this is challenging because they are highly transient and present in low abundance in wild-type cells at steady state.

A diverse range of methods has been used to measure the kinetics of RNA processing. One approach involves inhibiting RNA polymerase II (Pol II) activity with a transcription inhibitor such as 5,6-dichloro-1-β-D-ribobenzimidazole, then removing this inhibition to promote synchronized transcription. For example, measuring the delay after Pol II reaches the 3′ ends of introns before the spliced flanking exons can be detected provides an estimate of the time for splicing [8]. Splicing kinetics have also been estimated in human cells using fluorescence recovery after photobleaching and live cell imaging, by measuring either the amount of time that fluorescently tagged spliceosomal small nuclear ribonucleoproteins associate with transcripts, or the time taken for an intron-tethered green fluorescent protein-fusion protein to be removed by splicing [9]. By these diverse approaches, estimates of the time taken to splice introns in human cells have varied between 30 s and 5–10 min. For budding yeast, Alexander et al. [10] used an inducible reporter gene and high resolution kinetic analysis of RNA by quantitative reverse transcription polymerase chain reaction (RT-qPCR), estimating that splicing occurred within 30s of the production of the pre-mRNA. There are also computational approaches for predicting the speed of RNA processing events [11].

Metabolic labeling is a way of proportionally enriching classes of RNA that are difficult to detect in wild-type cells at steady state. For example, 4-thiouridine (4sU) has been used to label newly synthesized transcripts in human cells [12] and budding yeast cells [13] followed by biotinylation of the thiolated RNA to allow its selective recovery for microarray analysis. Neymotin et al. [14] used 4-thiouracil (4tU) labeling of RNA in budding yeast followed by RNA sequencing o determine RNA degradation rates, from which synthesis rates were derived.

Labeling with 4sU has been used to measure RNA synthesis, decay and splicing rates in human and yeast; however, the shortest labeling time was 3 min, by which time a substantial fraction of the newly transcribed RNA was already spliced or degraded [13, 15]. Therefore, to be able to measure RNA processing rates with higher accuracy and resolution transcriptome-wide, we have developed an extremely short (as little as 60 s) 4tU RNA labeling protocol and combined it with high-throughput RNA sequencing (RNA-seq). We demonstrate that our method (4tU-seq) readily detects low abundance and labile transcripts in wild-type cells that are normally detected only in cells that are defective in RNA degradation. Our data show that at such short times, lncRNA degradation kinetics depart significantly from first-order, and quantitatively associate lncRNA turnover with structural features of the transcripts. Also, using 4tU-seq we could, for the first time, measure relative pre-mRNA splicing kinetics transcriptome-wide in budding yeast. Unexpectedly, our results show that fast splicing of intron-containing ribosomal protein mRNAs largely depends on the degree of secondary structure between the 5′ splice site (5′ss) and branch point (BP) sequence and indicate that there is an optimal range of stability of intron secondary structures and base composition that allows for rapid splicing.

## Results and discussion

### Thiolated RNA can be efficiently recovered after very brief 4tU labeling

To isolate short-lived RNA species from the yeast *Saccharomyces cerevisiae*, we incubated cells with 4tU for very short periods, extracted the RNA, and treated it with a thio-reactive reagent to biotinylate the newly synthesized transcripts that contain thiol moieties [12, 16]. The biotinylated RNA was then affinity-purified with streptavidin. To improve 4tU incorporation during very short periods, the uptake of 4tU by yeast cells was enhanced by overexpressing the *FUI1* permease gene from a plasmid [17, 18]. Cell metabolism was rapidly halted by snap-freezing the labeled cells directly in very cold methanol, which is crucial for the recovery of short-lived RNAs [10]. Furthermore, each stage of the RNA isolation was carefully optimized to reduce background and maximize yield, in particular by using a modified binding and wash buffer (detailed in “Methods”). We used 4tU rather than 4sU for our studies because 4tU is much less expensive and gives very comparable incorporation rates (data not shown).

**Fig. 1:**
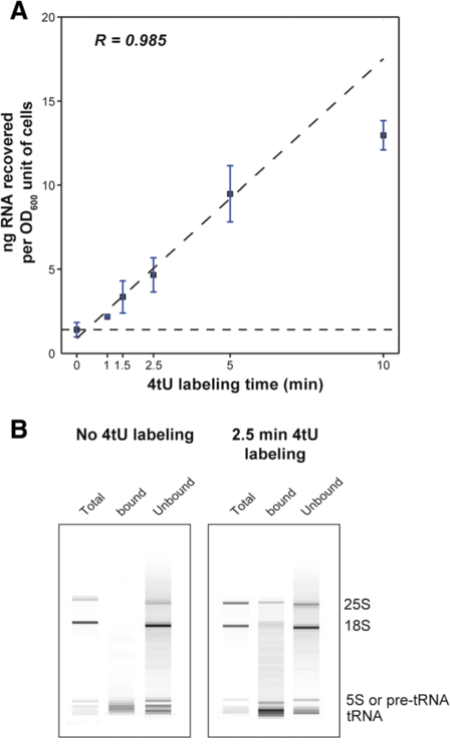
RNA yield increases with labeling time. **a** Plot of total yield of RNA recovered in nanograms per OD_600_ unit of cells against labeling time in minutes. During the first five minutes of labeling, yield increases linearly with labeling time (R^2^ = 0.985). Some RNA is recovered when 4-thiouracil (*4tU*) is not added to the culture (0 min). This is essentially background RNA that non-specifically bound to the magnetic beads during the isolation procedure. The *horizontal dashed line* indicates the level of background for this experiment. The actual amounts of RNA recovered from 600 ml culture (average of two experiments; R^2^ = 0.960) were: 0.50 μg (0 min), 0.77 μg (1 min), 1.16 μg (1.5 min), 3.30 μg (5 min) and 4.52 μg (10 min). **b** Agilent Bioanalyzer trace demonstrating the qualitative differences between the different fractions produced during the isolation of newly synthesized RNA. The *left panel* displays the results when no 4tU is added while the *right panel* shows 2.5 min labeling. The nonspecific background can be seen to be different from the total RNA comprising mostly short fragments. *tRNA* transfer RNA. 25S, 18S and 5S indicate mature ribosomal RNA species.

There was a linear increase in the yield of thiolated RNA over short labeling times up to 5 min, after which a component of the system became limiting (Fig. 1a; R^2^ = 0.99). Moreover, labeling for only 1.5 min was sufficient to achieve at least 2-fold enrichment over the background (yield from an unlabeled sample). By fitting a line to the data to indicate background levels, we deduced that the estimated time required before any 4tU was incorporated was about 30 s (Fig. 1a). Bioanalyzer analysis of mock samples indicated that most of the background consisted of short RNA species (Fig. 1b), which were mostly highly abundant tRNAs (Additional file 1: Figure S1).

### 4tU-seq proportionally enriches low stability species of RNA

We next performed RNA-seq on thiolated RNA (4tU-seq) isolated after 1.5, 2.5 and 5 min of 4tU labeling, on unlabeled control samples (i.e., “background”) and on rRNA-depleted total RNA, with all experiments performed at least twice. Additional file 1: Tables S1 to S14 provide all the results of our 4tU-seq data bioinformatics analyses. Additional file 1: Table S1 lists the total number of uniquely mapped reads for each sample. Notably, for the majority of RNA species we did not observe a significant correlation between the fraction of uridines in the transcript and the read coverage or RNA half-life (Additional file 1: Table S2 and Figure S2). However, this was not the case for snRNAs, which had a small sample size (only six) and are renowned for being U-rich (Additional file 1: Table S2).

**Fig. 2:**
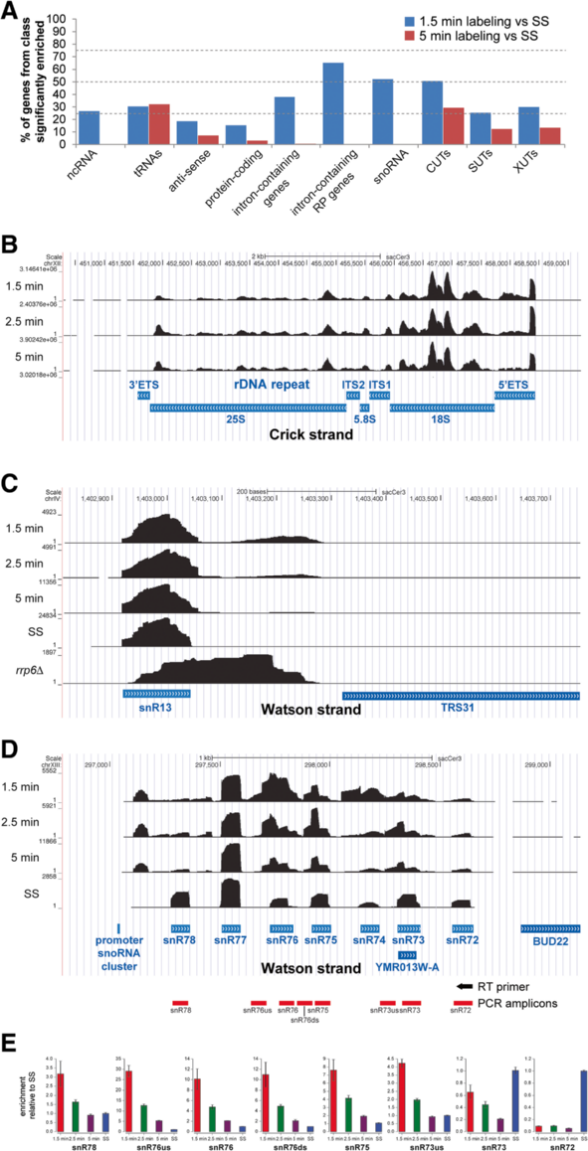
Short labeling times proportionally enrich unstable transcripts. **a** DESeq2 [19] was used to identify features significantly enriched in 4tU-seq data from short labeling times (1.5 min and 5 min) compared to total RNA. The figure displays the percentage of transcripts in each category that was found to be significantly enriched (DESeq2 adjusted *p* < 0.05). For the DESeq analyses, all reads were considered. Thus for the intron-containing mRNAs we used reads that mapped to both introns and exons. **b**-**d** UCSC genome browser screen shots showing the change in distribution of reads at different labeling times (Y-axis), with annotation below in *blue. SS* indicates steady-state levels, generated by sequencing total RNA. **b** 4tU detects pre-rRNA precursors. Note that the total RNA sample is not shown because it was rRNA depleted. **c** 4tU-seq detects 3′ extended snR13 species. Data from an rrp6Δ strain are displayed for qualitative comparison. **d** Polycistronic precursor from which multiple snoRNAs are processed. Blue boxes represent the annotated mature snoRNAs. **e** Real time (*RT*) quantitative polymerase chain reaction (*PCR*) validation of the 4tU-seq results shown in (**d**). For the RT reaction, a reverse transcriptase primer was used that was complementary to the 3′ end of the snR72 snoRNA. This cDNA was then used to amplify the different amplicons shown below each bar plot (see the illustration in (**d**) for what each amplicon represents). The data were then normalized to the results obtained with rRNA-depleted total RNA (SS). *5'ETS 5' external transcribed spacer, 3' ETS 3' external transcribed spacer*, *4tU* 4-thiouracil, *CUTs* cryptic unstable transcripts, *ITS internal transcribed spacer*, *ncRNA* non-coding RNA, *RP* ribosomal protein, *snRNA* small nuclear RNA, *snoRNA* small nucleolar RNA, *SUTs* stable unannotated transcripts, *tRNA* transfer RNA

We then used DESeq2 [19] to calculate the enrichment of different classes of RNA in the 4tU-labeled samples relative to the total RNA samples (see “Methods” for more details on the differential analyses of transcript abundance). In the 1.5-min 4tU-seq samples, a high proportion (37 %) of intron-containing transcripts were significantly enriched (adjusted *p* < 0.05; Fig. 2a), with substantially less enrichment seen in the 5-min samples. This is not surprising given that longer labeling times approach the steady state situation, where more of the labeled transcripts are spliced, such that the proportion of intron-containing transcripts is reduced. This illustrates the benefit of labeling for extremely short times. To our knowledge, 90 s is the shortest labeling period after which RNA-seq has been performed [14, 20].

Notably, many CUTs, SUTs and XUTs were significantly enriched in the 4tU samples relative to total RNA, with the degradation of these species appearing relatively slow compared to pre-mRNA splicing (discussed below).

Many non-coding RNAs (ncRNAs) are matured by cleavage and/or trimming of precursors at the 5′ and/or 3′ ends and the precursors were very well represented in our 4tU-seq data. Pre-ribosomal RNAs (pre-rRNAs) were readily detectable after 1.5 min of labeling, as judged by the accumulation of reads mapping to the external-transcribed and internal-transcribed spacer of the rDNA unit (Fig. 2b). The processing of these spacer regions seems to be slow given that they were still abundantly detectable after 5 min of labeling. It has been reported that incubation of human cells with concentrations of 4sU ≥50 μM over long periods of time (>1 h) causes a nucleolar stress response and inhibition of the production and processing of rRNA [21]. It is not known whether 4tU incubation for the very short times used here affects rRNA processing and, because rRNA was depleted from our total RNA samples before sequencing, we did not calculate relative pre-rRNA processing speeds.

The snR13 snoRNA is processed from a 3′ extended precursor [22] that accumulates in nuclear RNA surveillance mutants [23, 24]. Our 4tU-seq data suggest that most 3′ extended snR13 species are processed within 5 min of 4tU labeling. Another striking example from our 4tU-seq data was the rapid processing of snoRNAs from the snR72-snR78 polycistronic transcript [25] (Fig. 2d), which we confirmed by reverse transcriptase quantitative PCR (RT-qPCR; Fig. 2e). These results clearly demonstrate the potential of very short 4tU-labeling experiments for measuring the processing kinetics of short-lived RNA species.

### Degradation rates of cryptic transcripts correlates with transcript length and secondary structure

**Fig. 3:**
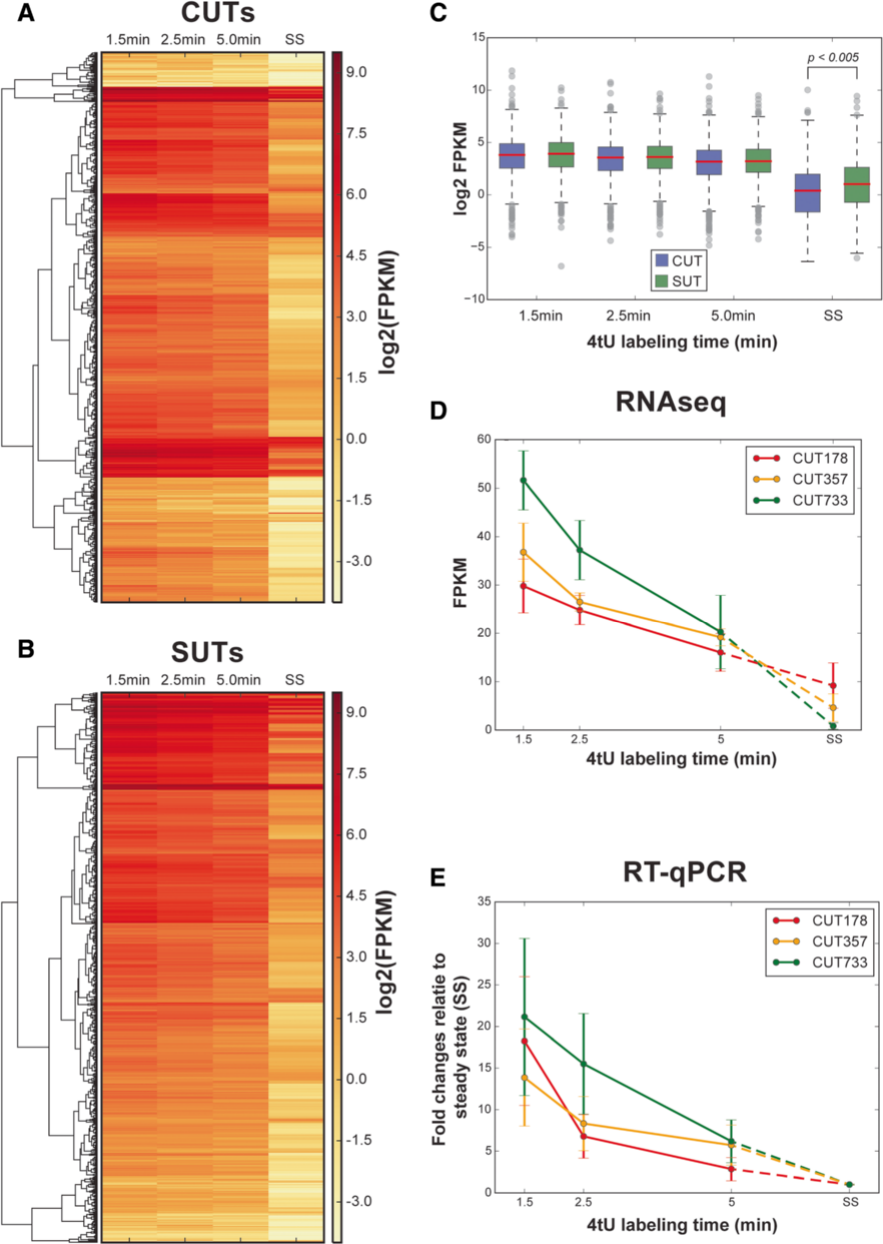
Cryptic unstable transcripts (*CUTs*) and stable unannotated transcripts (*SUTs*). **a** Heat map of fragments per kilobase per million reads (*FPKM*) at log2 scale for 887 CUTs at 1.5 min, 2.5 min, 5.0 min and steady state (*SS*). The hierarchical clustering is based on complete similarity between the FPKMs of two CUTs at the four time points. **b** The same heat map for 823 SUTs. **c** Comparison between log2 FPKM of 887 CUTs and 823 SUTs at 1.5 min, 2.5 min, 5.0 min and steady state. The levels of CUTs and SUTs are similar at initial time points but significantly different at steady states. **d** FPKM changes for three example CUTs measured by RNA-seq. **e** Levels of the same three CUTs relative to steady state, measured by reverse transcription quantitative polymerase chain reaction (*RT-qPCR*). *4tU* 4-thiouracil

A comparison of the enrichment of the different classes of cryptic transcripts for the 1.5 and 5 min labeling times relative to the total RNA samples indicated that CUTs, SUTs and XUTs were all readily detected and decayed more slowly than intron-containing transcripts and snoRNAs (Fig. 2a, Table 1, Additional file 1: Figure S1). The heat maps in Fig. 3a, b show hierarchically clustered normalized read data for 887 CUTs and for 823 SUTs [26] at 1.5 min, 2.5 min, 5.0 min and steady state. They clearly show the slow decay of both types of transcripts. The expression levels of CUTs and SUTs were generally similar at early labeling times but significantly different at steady states (*p* < 0.05; Fig. 3c). Interestingly, the levels of both CUTs and SUTs rose rapidly and remained approximately constant for the first 5 min at a level that was considerably higher than the steady state (Figs 2a and 3c, Additional file 1: Figure S1). This seems incompatible with the widely adopted first order kinetic assumption [27] and suggests that processing of these RNA species involves multiple steps, inducing delays that are comparable with the sampling times used in this study.

**Table 1:** Comparison of degradation dynamics between 925 cryptic unstable transcripts (*CUTs*) and 847 stable unannotated transcripts (*SUTs*)

Time gaps	CUT decrease	SUT decrease	*p*-value (t test)
1.5–2.5 min	0.31	0.32	0.79
1.5–5.0 min	0.63	0.68	0.84
1.5 min–steady state	3.41	2.86	3.9 × 10^−10^

The first column shows the gap between the two time points considered. The second column is the median decrease between the two time points for CUTs [computed as log2(fragments per kilobase per million reads)]. The third column shows the same quantity for SUTs. The fourth is the t-test *p*-values of the differences between CUTs and SUTs decreases.

The 4tU-seq data for three example CUTs (Fig. 3d) were compared with RT-qPCR analysis of the same three 4tU-labeled CUTs (Fig. 3e), and showed good agreement between the two methods. Thus, despite the fact that these RNA species are labeled as “unstable,” processing of many CUTs appears to be relatively slow.

**Fig. 4:**
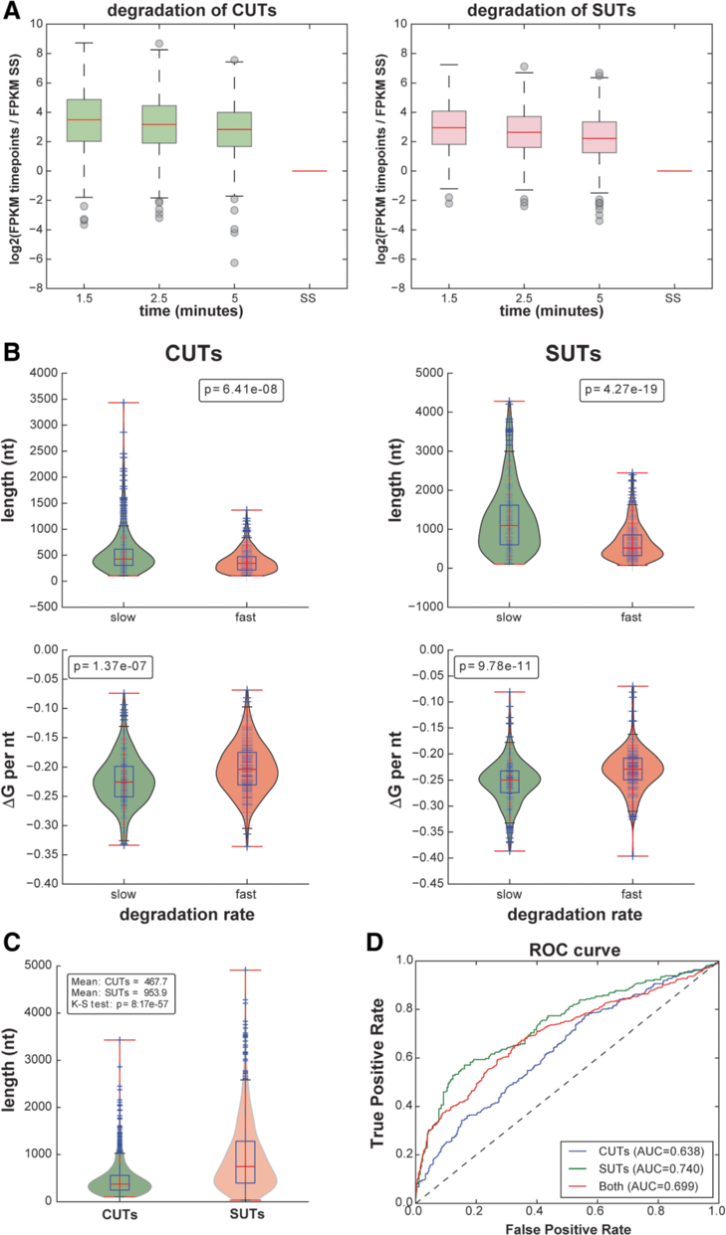
Analysis of degradation of cryptic unstable transcripts (*CUTs*) and stable unannotated transcripts (*SUTs*). **a** The log2 scaled ratio of CUTs fragments per kilobase per million reads (*FPKM*) normalized to the steady state (*SS*) levels. These ratios were used to quantify the degradation rate. A weighted average of the three nascent ratios was used to quantify the degradation rate, that is, the higher the ratio at the nascent points, the faster the degradation. **b** Comparison of features between the fastest-degrading third and slowest-degrading third of CUTs/SUTs. Features include average secondary structure free folding energy for each nucleotide, and transcript length. **c** SUTs are significantly longer than CUTs. Comparison of CUT and SUT transcript length distribution using the Kolmogorov–Smirnov (*K–S*) test. **d** The binary classification between the fastest-degrading third of transcripts and the slowest-degrading third of transcripts using ΔG per nucleotide (*nt*), transcript length, ΔG of ±15 nt around the start site and ΔG of ±15 nt around the stop site. The receiver operating characteristic (*ROC*) curves shows the data for the CUTs, SUTs or both with 10-folder cross-validation via a naive Bayes classifier. The area under the curve (*AUC*) is used to represent the prediction performance

We then sought to quantitatively explain the observed processing kinetics of CUTs and SUTs from sequence and structural features. As a measure of decay rate for each transcript, we considered the log ratio of the average expression level at the early times (1.5, 2.5 and 5 min) over the steady state expression level (Fig. 4a). The rationale behind this choice was the following: given the strong deviation from first order kinetics at early times, we assumed that early measurements were effectively proportional to RNA production rate, whereas the steady state expression levels were approximately equal to the ratio of production rate to decay rate (assuming that the initial deviation from first order kinetics becomes unimportant over long times). We observed that this decay rate was significantly negatively associated with transcript length and predicted secondary structure (Fig. 4b); in other words, long and/or highly structured transcripts were more stable. This is consistent with the model that many ncRNAs are subject to early termination by the Nrd1-dependent pathway and rapid degradation by the nuclear exosome [1–3, 4, 28] and/or are degraded in the cytoplasm (e.g., by Xrn1 [5]). Because SUTs are significantly longer than CUTs (Fig. 4c), our data provide a possible explanation for why SUTs are generally more stable than CUTs. We note that, in a regime where transcription is sporadic, transcript length could be expected to be positively associated with recovery at early time points, because longer transcription times would increase the probability of a transcript being labeled. Our analyses failed to reveal a significant effect of transcript length on the recovery of relatively short transcripts such as CUTs and SUTs at all time points (Additional file 1: Figure S3). Indeed, a bias toward longer transcripts at earlier labeling times would lead to a negative association of length with stability (longer transcripts would appear to have higher early expression and hence higher decay rate), which is the opposite of what we observed.

When attempting to fit a first order kinetic model to the data, the resulting degradation rates were uncorrelated to transcript length or structural features (data not shown), further underlying the inappropriateness of the first order assumption at these extremely short labeling times. Furthermore, using predicted secondary structure and transcript length alone, we were able to train a machine learning classifier to classify CUTs and SUTs with reasonable accuracy (Fig. 4d; area under receiver operating characteristic curve was 0.68 for CUTs and 0.73 for SUTs, random baseline 0.5).

### Very short 4tU labeling enables accurate measurements of splicing kinetics

In the case of intron-containing transcripts, the extent of RNA splicing can be determined by several alternative approaches [7], for example, from sequence reads that span intron–exon boundaries (splice sites) relative to reads that cross splice exon junctions [29], or from the number of intron reads relative to total gene reads [20]. Importantly, the transcription rate for any given gene should be constant during short periods of growth at steady state and will affect intron and exon production similarly, allowing relative splicing speeds to be determined by comparing pre-mRNA and exon levels at different labeling times without the influence of transcription rate. In vivo, spliceosome assembly occurs largely co-transcriptionally [7] and, theoretically, splicing catalysis could occur on a nascent transcript as soon as the 3′ss exits the polymerase. Additionally, because the 4tU-label can be incorporated at any position in the transcript at the time when the label is added, spliced mRNAs can be labeled even at the earliest time point. This could explain the very rapid splicing of some transcripts. Splicing also occurs in competition with pre-mRNA degradation [30, 31]; however, because degradation removes both the intron and the exon, this would not affect our estimates of splicing ratios.

In this work, we used a probabilistic model to estimate splicing ratios, that is, the proportion of spliced mRNA out of the total RNA for each transcript. This method was a modification of the MISO model [32] for quantification of splicing isoforms. Briefly, the method used information from all mapped reads by introducing a latent categorical variable for each read: the identity (i.e., whether it came from mature or precursor mRNA). The method then used computational statistical tools to compute a posterior probability of the identity of each read, therefore providing a quantification of mature and precursor mRNA. It is important to observe that in some cases (e.g., boundary or junction reads) the identity of a read is unambiguous, and indeed many direct methods only use unambiguously assigned reads; however, it was convincingly shown [32] that using information from all reads leads to more accurate estimations of isoform abundance. For more details, and for a comparison of different estimation methods, see Additional file 1: Data and Methods.

A major benefit of using a probabilistic model for estimating the abundance of precursor and mature mRNA lies in the possibility of obtaining posterior confidence intervals (CI) on the splicing ratios, which can then be used to filter noise in a principled way. In the analysis, we only retained genes with 95 % CI < 0.3, filtering out genes for which reliable estimation was not possible (primarily due to low sequence coverage). This filtering improved the correlation between replicates from 0.757 to 0.864 (see Additional file 1: Tables S3, S4 and S7). We also used a simulation to compare quantitatively the performance of the probabilistic model against the two direct methods that use the splice junction and intron boundary reads only, or intron reads and exon reads. The simulation showed that the probabilistic model yielded considerably lower variance results than the direct methods (Additional file 1: Figure S4), and therefore likely generated better results, especially when analyzing genes with low read coverage.

**Fig. 5:**
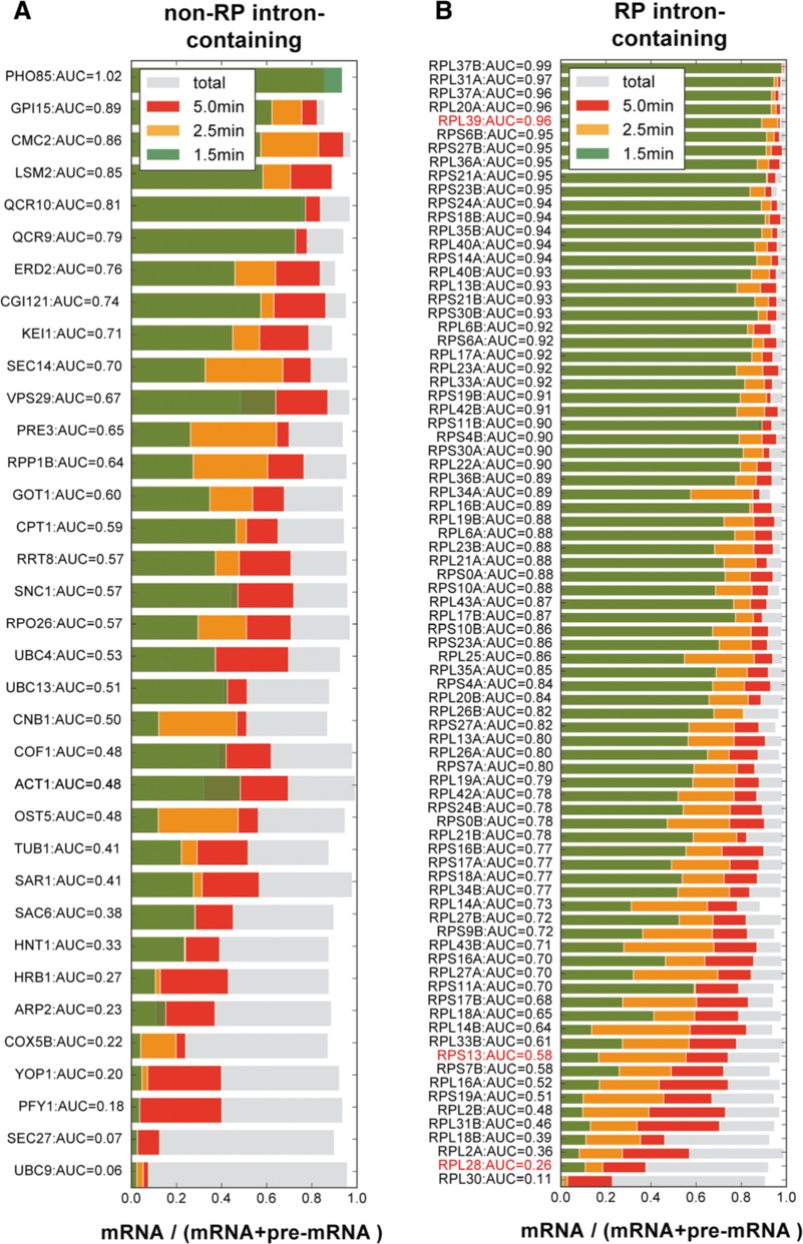
The splicing speed and associated features. The mRNA proportions at 1.5 min, 2.5 min, 5.0 min and steady state for 35 non-ribosomal protein (*RP*) intron-containing genes (**a**) and 82 RP intron-containing genes (**b**). The proportion of mRNA is estimated using the probabilistic model described in Additional file 1: Data and Methods RNA-seq data, and the area under the curve (*AUC*) score denotes the splicing speed as defined in the “Methods” section. Faster splicing transcripts cluster at the top, and slower splicing transcripts at the bottom. *Red* transcripts were also validated by reverse-transcription quantitative polymerase chain reaction (Fig. 6). All four colored bars are overlapping

In this 4tU-seq analysis, 187 intron-containing transcripts were selected that had a fragments per kilobase per million reads score (FPKM) of >10, that did not encode snoRNAs in the introns, and that only contained a single intron (to simplify the data analyses). Following posterior CI filtering, data for 82 ribosomal protein (RP) and 35 non-RP intron-containing genes were retained for the splicing ratio analysis (Additional file 1: Table S7). Figure 5 shows the mean splicing ratio of the three replicates at different time points for the 35 non-RP and 82 RP intron-containing genes, in which transcripts are ranked by speed of splicing from fastest (top) to slowest (bottom).

We also estimated the amount of background unlabeled (pre-)mRNA in our samples to determine to what extent this could influence our estimated splicing speeds. For these calculations we used RNA sequencing data generated from “background” or “0” samples (0 time point in Fig. 1a), which were derived from RNA that non-specifically bound to the magnetic beads during the isolation procedure (see “Methods” for details). We considered the intronic and exonic FPKM of the 250 intron-containing protein-coding genes in the background and 1.5-min samples. The middle column in Additional file 1: Figure S1 shows that the intronic FPKM percentages were 7.5 % and 46.1 % in the background and 1.5-min samples, respectively. Intuitively, the presence of background RNA will tilt this balance towards spliced mRNA, because unlabeled RNA is overwhelmingly exonic in the background samples (Additional file 1: Figure S1). Using these data, we estimated the background proportion using the difference between the 4tU-labeled intronic RNA fraction and the actually measured fraction. Theoretically, the intronic FPKM percentage in the signal should be lower than 50 %, with this bound being attained if spliced intronic RNA decayed at the same rate as mature mRNA (clearly a worst case scenario). Using this theoretical assumption, we estimated the upper bound on the background mRNA levels in the 1.5-min samples to be <9.1 % (see details in “Methods”). We conclude that this low level of mRNA background may have very slightly affected our kinetic estimates of splicing speeds but will not have affected the rankings (Fig. 5).

**Fig. 6:**
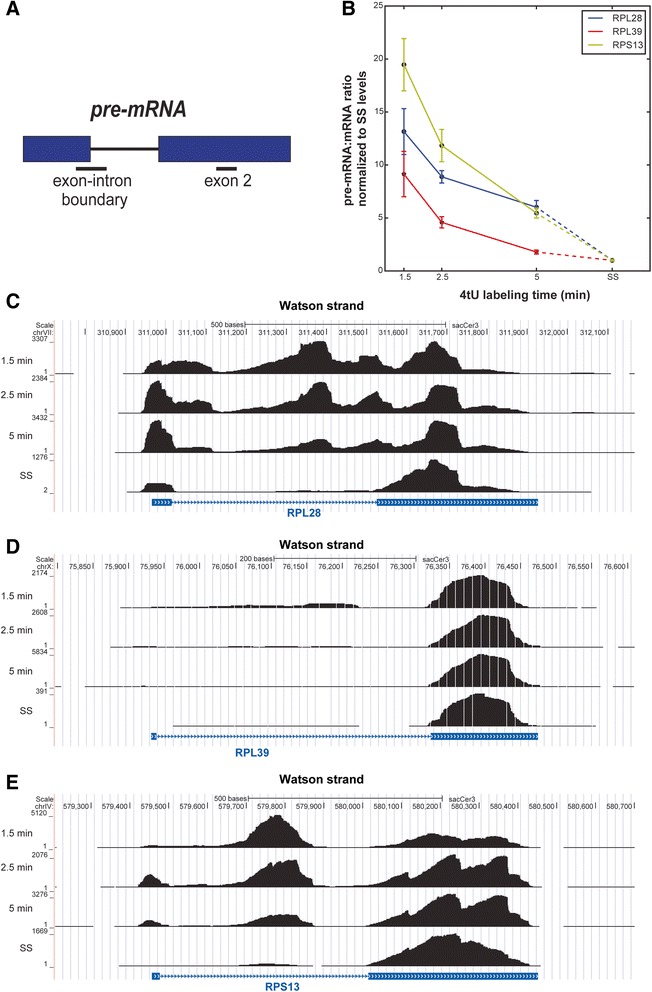
Reverse transcription quantitative polymerase chain reaction (RT-qPCR) analysis of splicing status shows differences between transcripts. **a** Diagram showing the location of diagnostic amplicons. Exons are denoted by *blue bo*xes and the intron is represented by a *black line*. Amplicons are indicated by lines below. Pre-mRNAs were detected using oligonucleotides that amplify the exon–intron boundary at the 5′ splice site. **b** Relative pre-mRNA levels of three transcripts, *RPL28, RPL39* and *RPS13*, analyzed by RT-qPCR, normalized relative to steady state (*SS*) levels. Data show how the level of each amplicon approaches the level detected at steady state as labeling time increases. Data were normalized to the levels of exon 2 and steady state to account for different RNA yields obtained at each labeling time. Different transcripts show different rates of splicing. **c**-**e** UCSC genome browser screen shots showing the change in distribution of reads at different labeling times (y-axis) for *RPL28*, *RPL39* and *RPS13*, with annotation below in *blue*. Exons are represented by *blue boxes* and intron indicated by a *blue line. SS* indicates steady-state levels, generated by sequencing total RNA

To validate some of our 4tU data, 4tU-RT-qPCR was performed on transcripts of three well-expressed ribosomal protein genes, *RPS13*, *RPL28* and *RPL39* (Fig. 6a, b). The levels of the 5′ss boundary amplicons (corresponding to unspliced pre-mRNAs) were normalized to the values for the corresponding exon 2 amplicon (Fig. 6a). The resulting values provided a measure of the proportion that corresponded to pre-mRNA. As the labeling time increases, the amount of exon 2 should remain more or less constant but the proportion of precursor should change. After 1.5 min of 4tU labeling, the proportion of pre-mRNA was high and decreased with labeling time, gradually approaching the level at steady state. The time required to reach steady state was transcript dependent, being approximately 5 min for RPL39, which was spliced relatively quickly, and longer for the others, in agreement with the rank order in Fig. 5b. These results confirm the enhanced recovery of unspliced pre-mRNAs by the 4tU-seq approach and its ability to detect different rates of pre-mRNA splicing for different transcripts. The results also validate the probabilistic model used.

One drawback of using intron read counts to measure splicing rates is that it is not always possible to distinguish between reads originating from pre-mRNA introns or intron lariats. This could potentially lead to an underestimation of splicing rates for some mRNA transcripts. However, based on the following results, we do not think that lariat reads significantly contributed to the splicing speeds reported here: several attempts were made to identify lariats in the 4tU-seq data using existing strategies [33] but only a handful of lariats could be detected. This is not surprising, because sequencing lariats tends to require use of *dbr1* mutants that lack lariat debranching activity and dedicated library preparation [33, 34]. Furthermore, we found a very high correlation between intronic read counts and counts for reads that overlap 5′ss–intron boundaries (Additional file 1: Figure S5). Therefore, we conclude that our use of intron versus exon reads accurately measures splicing rates.

### A/U richness and secondary structure of the intron affects ribosomal protein splicing kinetics

**Fig. 7:**
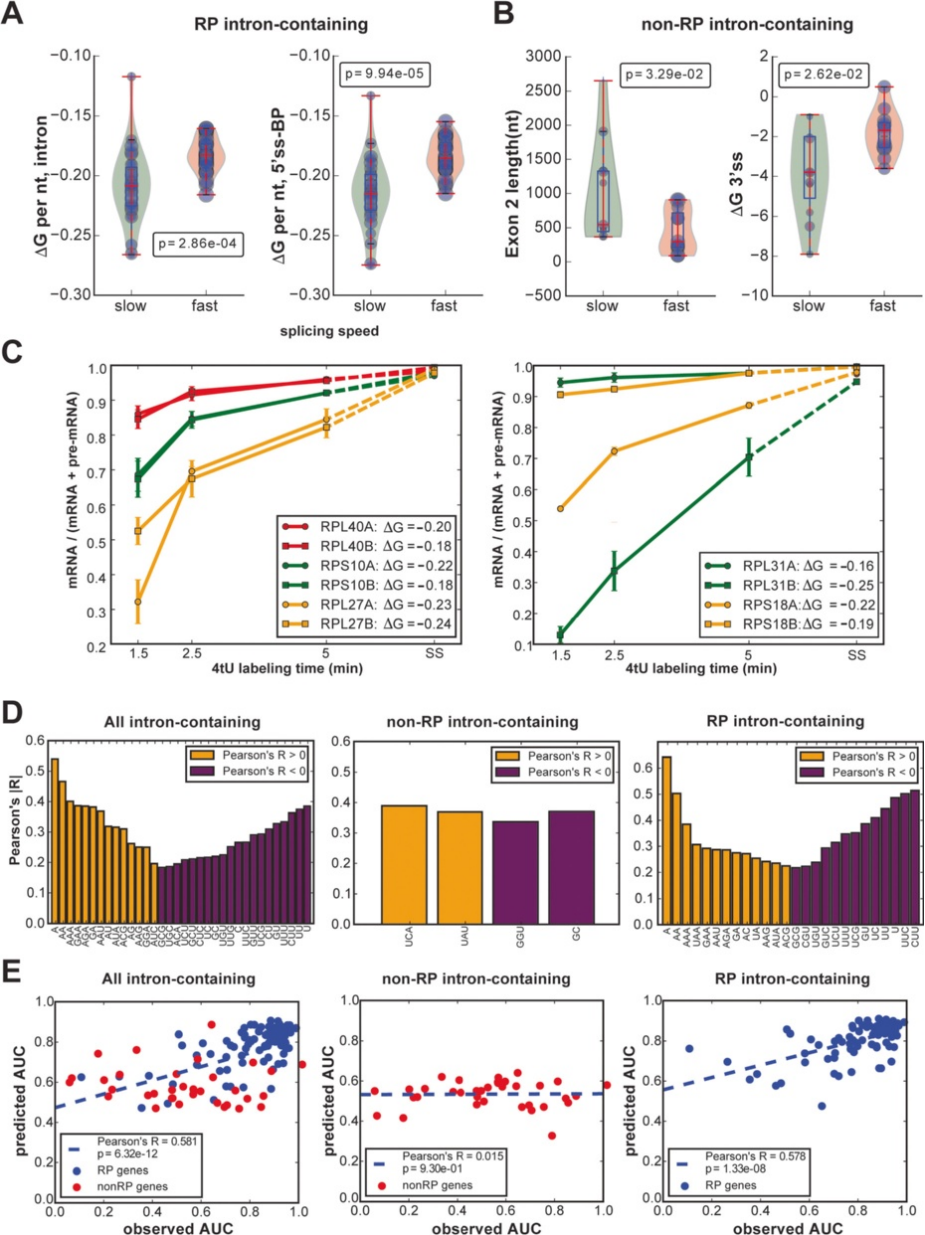
Features associated with splicing speed and comparison of paralogs. **a** Comparison of secondary structure scores (ΔG; y-axis) for the fastest-splicing and slowest-splicing thirds of 82 ribosomal protein (*RP*) intron-containing genes (x-axis). The violin plots show the distribution of the features, and the *blue dots* represent individual RP genes, with dot size corresponding to the splicing speed. The *p*-value was obtained using Wilcoxon’s test. **b** Comparison of exon 2 length and secondary structure at the 3′ splice site (3′ss) (y-axis) for 35 non-ribosomal protein (*non-RP*) intron-containing genes to splicing speed (x-axis). The violin plots show the distribution of the features, and the *blue dots* represent individual genes, with dot size corresponding to the splicing speed. The *p*-value was obtained using Wilcoxon’s test. **c** The mRNA proportion changes of three pairs of paralogs, each pair of which show a similar splicing rate (*left panel*) and of three pairs of paralogs, each pair of which shows different splicing rates (*right panel*). The proportion of mRNA is estimated using the probabilistic model described in Additional file 1: Data and Methods from 4-thiouracil (*4tU*) data. The ΔG per nucleotide values (see “Methods” section) between the 5′ss and branch point are stated in the inset boxes. **d** Pearson’s correlations between splicing speed and sequence patterns show the significantly correlated features (*p* < 0.05) to splicing speeds for all 117 intron-containing genes (*left panel*), 35 non-RP intron-containing genes (*middle panel*), and 82 RP intron-containing genes (*right panel*). The features are the occurrence of the specific base or bases in the intron. *Yellow* represents positive correlation with splicing speed and *purple* represents negative correlation. **e** Scatterplot of observed and predicted splicing speeds from the associated features. The features are listed in Additional file 1: Tables S8 and S9, and include secondary structures, splice site scores, intron length and exon length. The predictions are obtained by random forest regression with automatic feature selection. *AUC* area under the curve

Our results show that different introns were spliced at different rates based on area under the curve (AUC) calculations (see “Methods,” and scores in Fig. 5). Various features of introns could impact their speed of splicing. These include how close to consensus are the 5′ss, 3′ss and BP sequences as well as the strength of the secondary structure of the intron. We looked for correlations between different transcript features and the relative speed of splicing. Analyzing the fastest-splicing and slowest-splicing thirds of transcripts, we noticed there was a marked difference in the behavior of intronic RP transcripts compared to that of non-RP intronic genes: for the intronic RP transcripts, a highly significant difference (Wilcoxon's test *p* < 0.0003) was found only with regard to the normalized secondary structure scores of RP introns, with the major contribution coming from the 5′ss to BP region ( <1e04; see Fig. 7a). In the case of the non-RP transcripts, those that were spliced faster generally had less secondary structure at the 3′ss and a shorter exon 2 (Fig. 7b). All the feature comparisons are shown in Additional file 1: Figure S6 (non-RP transcripts) and Figure S7 (RP transcripts). The failure to see a significant effect of 5′ss, 3′ss and BP sequences in RP transcripts was likely due to the high similarity of these features.

The effect of intron secondary structure should be evident for paralogous RP genes that share highly related or identical exon sequences but have different intron sequences. Indeed, we found that some paralogs, such as RPL27A/B, RPS10A/B and RPL40A/B, that have introns with similar predicted secondary structure stabilities (ΔG values), had similar splicing speeds (Fig. 7c, left panel), whereas paralogs with introns that have different predicted ΔG values, such as RPL31A/B and RPS18A/B, had different splicing speeds (Fig. 7c, right panel).

Because the predicted secondary structure within RP introns was significantly correlated with the splicing speed, we further explored the intron sequences and found that several short base combinations were significantly correlated with splicing speed (Fig. 7d). Generally, fast splicing introns were enriched for adenosines, while slower splicing introns had a higher density of uridines. Surprisingly, the proportion of “A” and “U” in the introns was highly negatively correlated (Pearson’s correlation coefficient < −0.75). Furthermore, we used the above features (all listed in Additional file 1: Tables S8 and S9) to predict the splicing speed by a random forest regression model. Figure 7e shows that the splicing speeds for RP pre-mRNAs could be well predicted by these features (Pearson’s R = 0.578 between observed and predicted splicing speed).

Overall, the most significant features we have been able to identify that distinguish intron splicing rates for RP pre-mRNAs in budding yeast are the predicted secondary structure in the region between the 5′ss and the BP, and A or U density. In our data, slower splicing was associated with greater predicted secondary structure stability and U-richness in the intron. The effect of secondary structure was observed to be strongest for the set of highly expressed RP transcripts whose introns were mostly longer than average in budding yeast. The idea that secondary structure may have a role in splicing is not new [34–37], but previous work has mostly suggested that secondary structure in long introns in fact favors efficient splicing. Structure between the 5′ss and the BP has been proposed to be necessary for efficient splicing when this distance is greater than 200 nucleotides [37]. Work done on *RPS17B* by the Rosbash laboratory experimentally identified two complementary regions between the 5′ss and the BP that base paired to form a stem loop, thereby reducing the effective distance [38]. Mutations that disrupted this stem loop reduced the efficiency of splicing; compensatory mutations that restored the loop restored splicing efficiency. Further work by Rogic et al. [37] suggested that a specific structural arrangement was not required but that a very thermodynamically stable structure could slow splicing, possibly by masking splicing signals. Taken together with the results of Rogic et al. [37], our work indicates that efficient splicing of RP pre-mRNA transcripts requires an optimal amount of secondary structure between the 5′ss and the BP, with either too much or too little being detrimental. Furthermore, our results suggest that in the endogenous context, RP transcripts that splice slower are more often victims of too much structure rather than too little. A recent paper showed that fast splicing of reporter constructs correlated with low secondary structure around the splice sites [39]. For the RP genes we did not find a significant correlation between splicing speed and folding energies of splice sites (Additional file 1: Figure S7); However, this is presumably because the splice signals for these RP genes are very similar to the consensus sequences. We did, however, find a correlation between weak folding of the 3′ss sequence and efficient splicing for the non-RP genes analyzed here (Additional file 1: Figure S6), consistent with previous work [39].

## Conclusions

4tU-seq is a powerful way of examining RNA processing kinetics and has many other potential applications. Our analysis at very short labeling times reveals unexpected complexity in RNA processing for several different families of RNAs. For lncRNAs, such as CUTs and SUTs, our data show that early abundances cannot be explained by simple first order kinetics, and quantitatively relates degradation of CUTs and SUTs to transcript length and predicted secondary structure.

Our analysis shows that to measure splicing kinetics in *S. cerevisiae* it is essential to recover the RNA after extremely short 4tU labeling times; many transcripts approach steady state by about 2.5 min. By measuring the speed of splicing of many different newly synthesized pre-mRNAs we were able to search for factors that could explain why some splice faster than others in vivo. For the 35 non-RP intronic genes for which we had adequate sequence coverage, we found a significant correlation between the secondary structure around the 3′ss and exon 2 length. This suggests that for these transcripts, selection of the 3′ss might be the rate-limiting step because it requires unwinding of RNA secondary structures. Differences were found even between intronic RP transcripts that have similar expression levels, the same gene annotation (Gene Ontology term), and similar lengths and strength of splice sites. Moreover, transcripts of certain highly related paralogous genes displayed differences in splicing speeds. Our results suggest that this difference in splicing kinetics is in part due to the secondary structure of the introns as well as the nucleotide composition. Within the RP gene subgroup, introns with a less favorable predicted secondary structure (less negative ΔG) spliced faster than those with more structure. One simple explanation for the observed trends is that it is more difficult for spliceosomes to assemble on introns with a stronger secondary structure and so more time is required to overcome this impediment. Another possibility is that structural re-arrangements required within the spliceosome to allow catalysis are costlier when there is more structure to contend with. Whether a transcript splices quickly or slowly carries over into its mRNA level at steady state, and it seems probable that regulating the secondary structure of the intron will in effect regulate the expression of the gene. Furthermore, in organisms with multi-intron genes and alternative splicing, an optimal degree of secondary structure could contribute to determining which introns get spliced in kinetic competition. It will be interesting to use 4tU-seq to study the kinetics of splicing under different metabolic and environmental conditions and to test the effects of different splicing factors on the speed of splicing.

## Methods

### Yeast strains and plasmids

The *ura3* point mutation in W303 (MAT**a**, ade2-1, ura3-1, his3-11, 15 trp 1-1, leu2-3, 112 can1-100) was corrected to create W303U. W303U was transformed with plasmid pAT1 (*FUI1* on the plasmid pRS425).

### 4tU-seq

Cultures were grown in Synthetic Defined –Ura –Leu medium to OD_600_ = 0.8, at which point 4tU was added to a final concentration of 500 μM. Cultures were maintained at 30 °C and were shaken throughout the experiment. Large (600 ml) cultures were used for 4tU-seq experiments. After the desired labeling time the cells were snap frozen by dropping the culture into a half volume of methanol in a large beaker sitting in dry ice to rapidly halt metabolism [10]. While the methanol slurry was still liquid, the frozen cells were pelleted by centrifugation at 3000 g for 3 min at 4 °C. RNA was isolated using a standard hot-phenol extraction. Biotinylation was performed on 2 mg of RNA as previously described [20], but only incubated for 15 min at 65 °C to reduce RNA degradation. The RNA was purified using 2 ml Zeba columns (ThermoFisher Scientific, Perth, UK), as per manufacturer's instructions, and ethanol precipitated. Biotinylated RNA was extracted using streptavidin C1 Dynabeads (ThermoFisher Scientific). We equilibrated 50 μl of beads in Na_3_PO_4_TMgCl_2_ (0.2 M NaCl, 0.1 M sodium phosphate pH 6.8, 25 mM MgCl_2_ and 0.4 % Tween) then blocked in Na_3_PO_4_TMgCl_2_ and 1 μg/μl glycogen for 20 min. The biotinylated RNA was purified for 30 min with the Dynabeads at 4 °C in Na_3_PO_4_TMgCl_2_. The beads were washed three times in Na_3_PO_4_TMgCl_2_ and twice with TEN1000 (10 mM Tris-HCl pH 7.5, 1 mM EDTA pH8 and 1 M NaCl). All bead block, incubation and wash volumes were 400 μl. The RNA was eluted twice with 50 μl of 0.7 M beta-mercaptoethanol for 5 min at room temperature before being ethanol precipitated with 20 μg glycogen at −20 °C for at least 2 h and re-suspended in 10 μl DEPC-treated H_2_O. To generate the 0 min or background RNA samples, we applied this protocol to cultures to which no 4tU had been added, which essentially provided an overview of RNAs that non-specifically bound to the streptavidin magnetic beads during the isolation procedure.

RNA-seq libraries were produced essentially as described previously [40]. Briefly, between 100 and 250 ng of (thiolated) RNA was fragmented in SuperScript Reverse Transcriptase buffer (ThermoFisher Scientific) for 5 min at 95 °C. Fragmented RNA was subsequently randomly primed as described [40]. Edinburgh Genomics (Edinburgh, UK) performed the 100-base pair paired-end sequencing using the Illumina HiSeq 2500 platform.

### Differential analysis of transcript abundance

To determine what classes of transcripts were significantly enriched between two time points (1.5 and 5 min) and in the total RNA (Fig. 2a), we used DESeq2 [19]. Three biological replicates of each time point were used for these analyses. For each comparison, DESeq2 generated a list of transcript names (such as CUTs, SUTs and protein-coding genes) that were significantly over-represented in each time point. We used a *p*-value of 0.05 as threshold. We then counted the total number of transcripts from each class over-represented at each time point and divided that number by the total number of transcripts in each class. This showed us what fraction of each class was significantly enriched in the thiolated RNA.

### Estimation of mRNA background levels in 4tU-seq data

To estimate the mRNA background levels in 4tU-seq data, we let the measured intronic FPKM percentage be *α*_0_ and *α*_1_ at 0 (“background”) and 1.5 min, respectively. We let the intronic FPKM percentage be *β* for signal; this value was unknown but we know from theoretical considerations that *β* ≤ 0.5. We denoted the background and the signal fractions of RNA mapped to intron-containing genes at 1.5 min as *m* and *n* respectively. This gave us *α*_0_ × *m* + *β* × *n*)/(*m* + *n*) = *α*_1_, so:

$$ m/n=\left(\beta -{\alpha}_1\right)/\left({\alpha}_1-{\alpha}_0\right)\le \left(0.5-{\alpha}_1\right)/\left({\alpha}_1-{\alpha}_0\right) $$

Therefore, the proportion of background reads over the total was bounded by [(*m*/*n*)/(1 + *m*/*n*)]. Substituting the observed values for *α*_0_ and *α*_1_, we obtained the 9.1 % upper bound as reported in the main text.

### RT-qPCR

RT-qPCR was carried out as described previously [10] using oligonucleotides listed in Additional file 1: Table S5.

### Processing of raw sequencing data

Sequencing was performed on a HiSeq 2500 by Edinburgh Genomics. Raw fastq files were demultiplexed using pyBarcodeFilter version 2.3.3 from the pyCRAC tool suite, version 1.2.2.4 [41]. Quality trimming and removal of 3′ adapter sequences (5′- AGATCGGAAGAGCACACG-3′) was performed using Flexbar, version 2.4 [42]. RNA-seq reads were aligned to the yeast genome by STAR [43]. Counts for annotated genomic features were generated using pyCRAC [41], in-house python scripts and genomic feature files (GTF) from ENSEMBL, version R64-1-1.75. Coordinates for CUTs and SUTs were obtained from Xu et al. [26]. The python scripts used for this study are available upon request.

### Quantification of splicing ratio and splicing speed

The splicing ratio, that is, the mRNA proportion, was then calculated from the aligned reads either by direct methods or using a probabilistic method (see Additional file 1: Data and Methods); the code for the probabilistic method is available at https://github.com/huangyh09/diceseq. To combine the measurements of pre-mRNA and mRNA abundance at 1.5, 2.5 and 5 min into a single measure of splicing speed, we considered the AUC as follows:

1 $$ AUC=\frac{\left({R}_{2.5}+{R}_{1.5}\right)/2+2.5\times \left({R}_{5.0}+{R}_{2.5}\right)/2}{3.5\times {R}_{SS}} $$

where *R*_*i*_ is the splicing ratio, that is, mRNA/(mRNA + pre-mRNA), at time point *i* (in minutes), estimated as the average of the posterior mean for the three replicates. *R*_*SS*_ represents the merged splicing ratio at the steady state. Therefore, the larger the AUC, the more efficient the splicing process.

### Intron-containing RP gene annotation and features

The lengths and sequences of various features were obtained from SGD (http://www.yeastgenome.org/). BP locations were obtained from the Yeast Intron Database [44]. The scores assigned to the 5′ss, 3′ss and BS were obtained using the method described by Crooks et al. [45]. The free-energy of the predicted secondary structure was calculated using quickfold (http://mfold.rna.albany.edu/?q=DINAMelt/Quickfold). This value was then divided by the number of bases in the intron (or part of the intron) to give the secondary structure per base (a ΔG value and therefore negative). The more negative the ΔG value, the more structured the intron is predicted to be.

### Data access

Raw (fastq) and processed sequencing data can be downloaded from the NCBI Gene Expression Omnibus repository [GEO: GSE70378]. All raw read count and feature data can be found in Additional file 1: Tables S6 to S14.

## Ethical approval

No ethical approval was required for this study.

## Additional file

Additional file 1:
**Tables S1–S14; figures S1–S7; and Supplementary Methods and** **References.** (ZIP 6028 kb)

## Abbreviations

3′ss: three prime splice site in intron
4tU-seq: 4-thiouracil labeling and sequencing
5′ss: five prime splice site in intron
BP: branch point sequence
CI: confidence interval
CUT: cryptic unstable transcript
ETS: external transcribed spacer
FPKM: fragments per kilobase per million reads
ITS: internal transcribed spacer
lncRNA: long non-coding RNA
mRNA: messenger RNA
miRNA: microRNA
ncRNA: non-coding RNA
non-RP: non-ribosomal protein
RNA-seq: RNA sequencing
RP: ribosomal protein
siRNA: small interfering RNA
snRNA: small nuclear RNA
snoRNA: small nucelolar RNA
SS: steady state
SUT: stable unannotated transcripts
tRNA: transfer RNA
XUT: Xrn1-dependent unstable transcript
