# Supplementary material for: Transcriptome-wide RNA processing kinetics revealed using extremely short 4tU labeling
Source: Genome Biol. 2015 Dec 17;16:282. doi: 10.1186/s13059-015-0848-1 (PMC4699367; doi:10.1186/s13059-015-0848-1)
Supplement: Additional file 1: — Tables S1–S14; figures S1–S7; and Supplementary Methods and References. (ZIP 6028 kb) [file 13059_2015_848_MOESM1_ESM.zip › Barrass et al Additional Data and Methods.pdf]

Transcriptome-wide RNA processing kinetics revealed using extremely short 4tU labeling

Additional data and methods

J David Barrass, Jane E A Reid, Yuanhua Huang, Ralph D Hector, Guido Sanguinetti,

Jean D Beggs and Sander Granneman

This file contains the following additional information:

Supplementary Tables S1 to S5

Supplementary Figures S1 to S7

Supplementary Methods

Supplementary References

Supplementary Tables

Table S1. Total number of uniquely mapped reads in the sequencing data. SS indicates steady-state RNA. Note that rRNA was depleted from the SS samples before sequencing.

Replicate	background	1.5 min	2.5 min	5.0 min	SS
1	31,857,677	10,438,022	12,726,794	9,224,517	4,215,612
2	10,870,860	13,306,188	12,732,443	21,927,409	57,277,471
3		28,898,557	22,403,922	25,711,226	18,823,326

Table S2. Pearson correlation between the percentages of uridines in RNAs and RNAseq FPKM values. Background numbers were generated from total RNA samples that were not 4tU-labeled. SS indicates steady-state RNA. Note that rRNA was depleted from the SS samples before sequencing.

Biotype	Size	background	1.5 min	2.5 min	5 min	SS
**CUTs**	925	-0.049	-0.101	-0.112	-0.095	-0.053
**SUTs**	847	-0.086	-0.044	-0.085	-0.087	-0.118
**XUTs**	1658	-0.092	-0.091	-0.098	-0.095	-0.073
**anti-sense**	402	-0.141	-0.127	-0.11	-0.101	-0.136
**ncRNA**	15	-0.302	-0.315	-0.315	-0.31	-0.302
**protein_coding**	6785	0.045	0.058	0.058	0.053	0.047
**pseudogene**	21	-0.112	-0.182	-0.126	-0.12	-0.048
**rRNA**	20	-0.078	-0.134	-0.167	-0.128	-0.033
**snRNA**	6	0.644	0.757	0.693	0.71	0.755
**snoRNA**	77	-0.177	-0.136	-0.095	-0.083	-0.109
**tRNA**	302	0.024	-0.028	0.008	0.08	0.198

Table S3. Spearman's correlation coefficients of splicing ratios or speed between replicates for 187 intron-containing genes. The splicing ratio is estimated by MCMC sampler and the AUC is the denormalized area under the curve of the three time points at 1.5, 2.5 and 5 minutes.

Items	Replicates 1 and 2	Replicates 1 and 3	Replicates 2 and 3
**Ratio: 1.5 min**	0.895	0.799	0.792
**Ratio: 2.5 min**	0.909	0.778	0.757
**Ratio: 5.0 min**	0.937	0.770	0.819
**AUC: raw**	0.939	0.867	0.858

Table S4. Spearman's correlation coefficients of splicing ratios or speed between replicates for 150 filtered intron-containing genes. Those genes have at least two replicates with 95% confidence interval < 0.3 for estimation of splicing ratio. The splicing ratio is estimated by MCMC sampler and the AUC is the denormalized area under the curve of the three time points at 1.5, 2.5 and 5 minutes.

Items	Replicates 1 and 2	Replicates 1 and 3	Replicates 2 and 3
**Ratio: 1.5 min**	0.914	0.886	0.876
**Ratio: 2.5 min**	0.926	0.878	0.861
**Ratio: 5.0 min**	0.943	0.864	0.871
**AUC: raw**	0.945	0.920	0.908

Table S5. Oligonucleotides used for this study

PCR	Oligo	Sequence (5'-3')
RPL28 5'SS	F	TTGGTTCTTTCATTCCCTCTTCCA
	R	TCCAGATTCACTAAGACTAGAAAGCACAGA
RPL28 exon2	F	AGAGGTATGGCCGGTGGTCA
	R	CAGAAATGAGCTTGTTGCTTGTGG
RPL39 5'SS	F	AACACAGATAGATCAACATGGCTGTATGT
	R	GGTGGTAAGGTCATTTAGATGGATGTG
RPL39 exon2	F	AGCAAAACAGACCATTGCCACA
	R	TGTTCATCTTGGTTCTTCTCCAGTTTC
RPS13 5'SS	F	TCGTATGCACAGTGCCGTATGTT
	R	TGATTTAGCGAACTATTCAATGCAACTTT
RPS13 exon2	F	CTAGAAATGCTCCAGCTTGGTTCAA
	R	TCAAACCCTTTCTCGCGTACTTG
CUT178	F	AAGGCTTGAGCCAGGGTTTGA
	R	TGGCGTGTTGACATTGGGTTT
CUT357	F	TCTTTCCTTTTCGCCCCAAAC
	R	GAACGCAGAGCTCAGGTGGAC
CUT733	F	AGGCAGTCCTTGTGGGTAAAGTTG
	R	GCCGACTGGTCCACAGTTGTT
snR72	F	TGATGACAACTTCTTGAGCTATATATTTTCTTGA
	R	CTTGTGATCAGACTGACGTGCAAA
snR73	F	CACGACGGTCAACTGCGTTT
	R	GCTCAGTACCACGCCCTGTCA
snR73us	F	GATGAGAGATCGTACAAACTGTTTCAAGGT
	R	AACGCAGTTGACCGTCGTGA
snR75	F	GAGATATTTATAGATGACGAGTCTGATCATTCG
	R	ACAGCTTCAGAAAGGCATGAATGG
snR76ds	F	CAATGCTTTAGCGGGCCTAGAA
	R	TTCCCTATAGAAAATTCTGTTAGCCCTCA
snR76	F	CACAGCACTGGGCACTGAGG
	R	GATCAGTTGCGCGGGTTTTC
snR76us	F	AGTTGACACATTTCCATAAATTGGAAGAA
	R	GATCGAAAACATCAATCTACGGAAGAA
snR78	F	CCTTGATGACCAAAATAAATTTTTACAAACTAGA
	R	CCTCAGAAATAAGAATAAACGTTCTAATCACAAA
Biotin Oligo		[5' Biotin] AACGGAAAGCTAGTCTTGCATTTCCGTG CTGGAGATCAGGATTTTGTGAGGCTCTTCTACTCCTTTT TCAAG TCCCTTGTGGGCAAGGATGTAGTCGTGG

Table S6. Read counts and FPKMs for all genomic features

Table S7. Read counts, mRNA proportion and 95% confidence intervals from all 4tU-seq libraries. "Ratio" indicates the proportion of mRNA, which was calculated by dividing mRNA over (mRNA+pre-mRNA).

Table S8. Intron and exon data used for identifying sequence and structural features that correlate with slow or fast splicing.

Table S9. Intron and exon data used for identifying sequence and structural features that correlate with slow or fast splicing.

Table S10. Intron, exon, junction and boundary reads FPKM from the background sequencing data.

Table S11. Intron, exon, junction and boundary reads FPKM from the 1.5 minute 4tU sequencing data.

Table S12. Intron, exon, junction and boundary reads FPKM from the 2.5 minute 4tU sequencing data.

Table S13. Intron, exon, junction and boundary reads FPKM from the 5 minute 4tU sequencing data.

Table S14. Intron, exon, junction and boundary reads FPKM from the rRNA depleted total RNA sequencing data.

Supplementary Figures

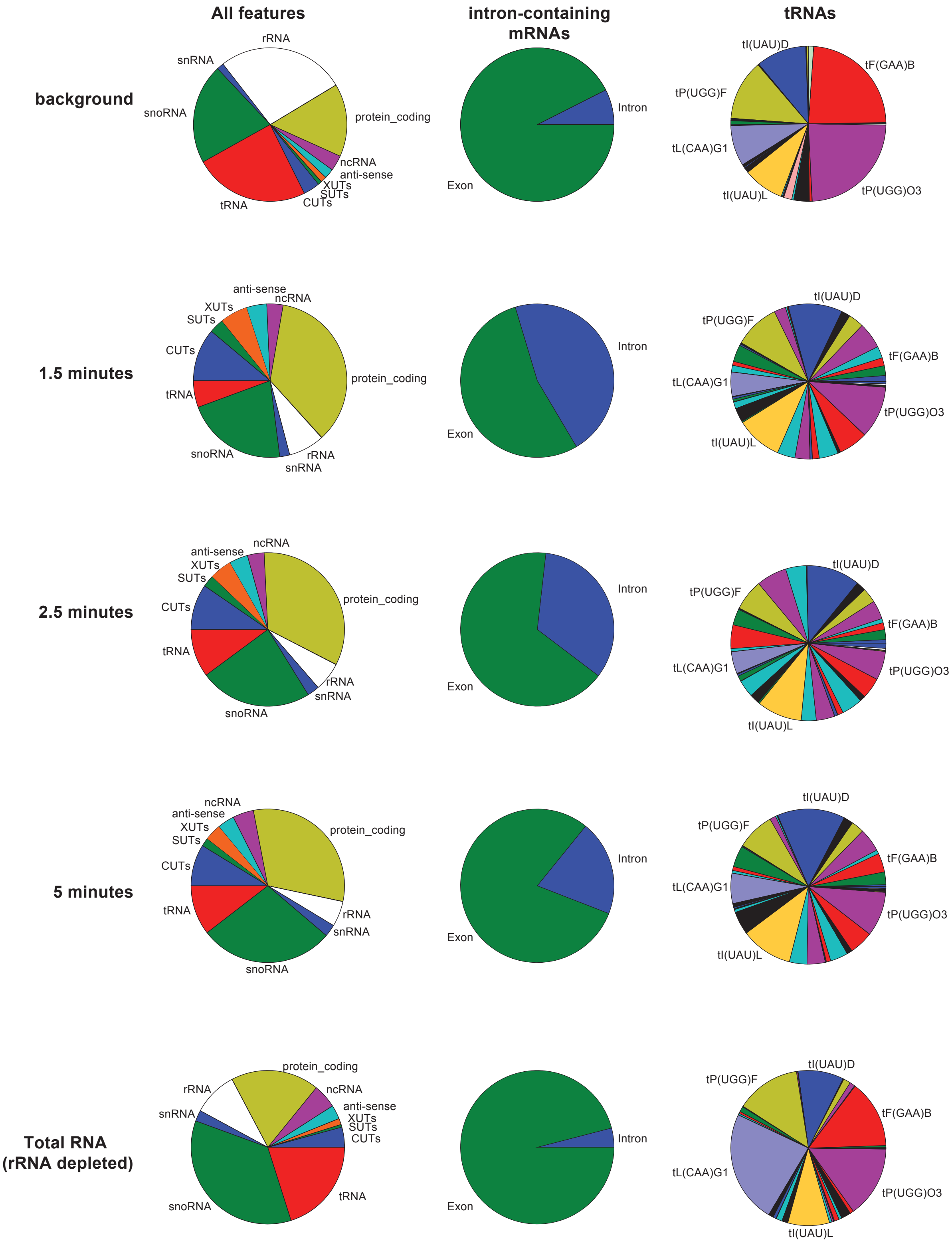

Figure S1. Pie charts showing the normalized read count distribution (FPKM) over all annotated genomic features, introns and exons and tRNAs. The pie charts shown in the middle show the distribution of intron and exon FPKM for 250 intron-containing protein-coding genes.

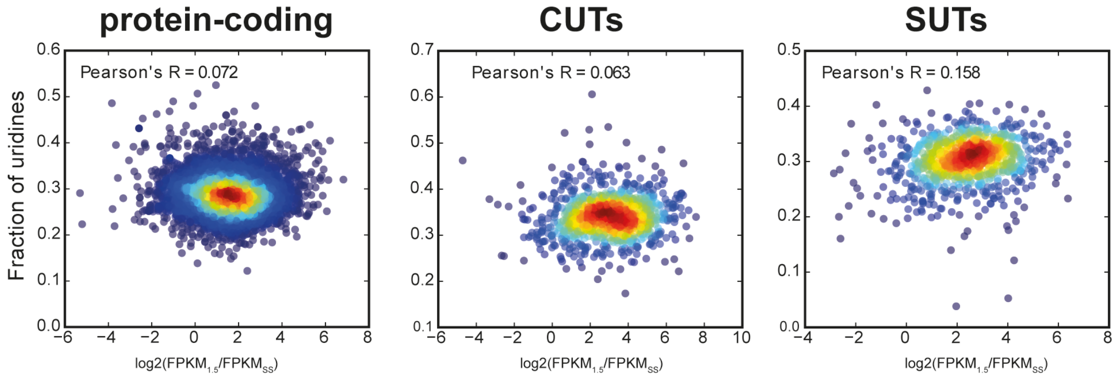

Figure S2. Uridine content of transcripts does not significantly correlate with RNA half-life. The scatter plots show fraction of uridines (y-axis) plotted against RNA half-life, which was calculated as FPKM of thiolated RNAs isolated after 1.5 minutes of labeling, divided by the FPKM of steady state RNAs. Pearson's R indicates Pearson's correlation coefficient.

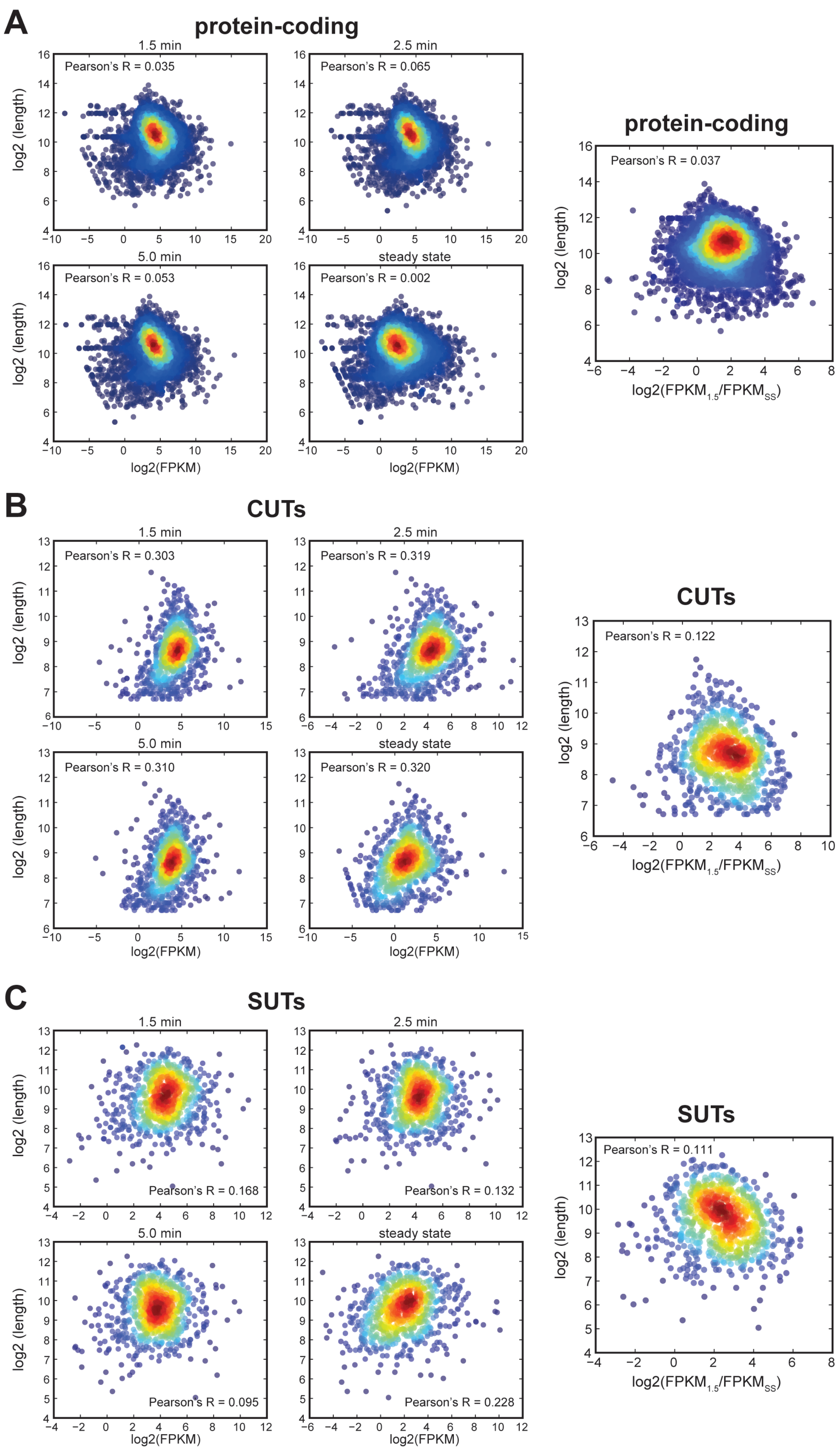
A
protein-coding
1.5 min
Pearson's R = 0.035
2.5 min
Pearson's R = 0.065
5.0 min
Pearson's R = 0.053
steady state
Pearson's R = 0.002
log2 (length)
log2(FPKM)
protein-coding
Pearson's R = 0.037
log2 (length)
log2(FPKM1.5/FPKMSS)
B
CUTs
1.5 min
Pearson's R = 0.303
2.5 min
Pearson's R = 0.319
5.0 min
Pearson's R = 0.310
steady state
Pearson's R = 0.320
log2 (length)
log2(FPKM)
CUTs
Pearson's R = 0.122
log2 (length)
log2(FPKM1.5/FPKMSS)
C
SUTs
1.5 min
Pearson's R = 0.168
2.5 min
Pearson's R = 0.132
5.0 min
Pearson's R = 0.095
steady state
Pearson's R = 0.228
log2 (length)
log2(FPKM)
SUTs
Pearson's R = 0.111
log2 (length)
log2(FPKM1.5/FPKMSS)

Figure S3. Length of transcript does not significantly with the transcript expression level or RNA half-life. The scatter plots show the log2 of transcript length plotted against the expression levels of the transcript (log2(FPKM)) or RNA half-life for protein-coding (A), CUTs (B) and SUTS (C). RNA half-lives were calculated as FPKM of thiolated RNAs isolated after 1.5 minutes of labeling, divided by the FPKM of steady state RNAs. Pearson's R indicates Pearson's correlation coefficient.

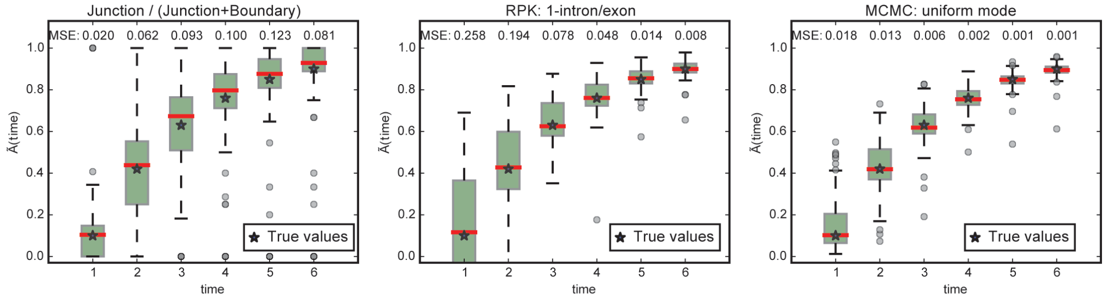

Figure S4. The estimated splicing ratios for 187 yeast intron-containing transcripts by three methods. The asterisks indicate the true values, and the mean squared errors (MSE) between 187 transcripts and the true values for each of the six time points are shown.

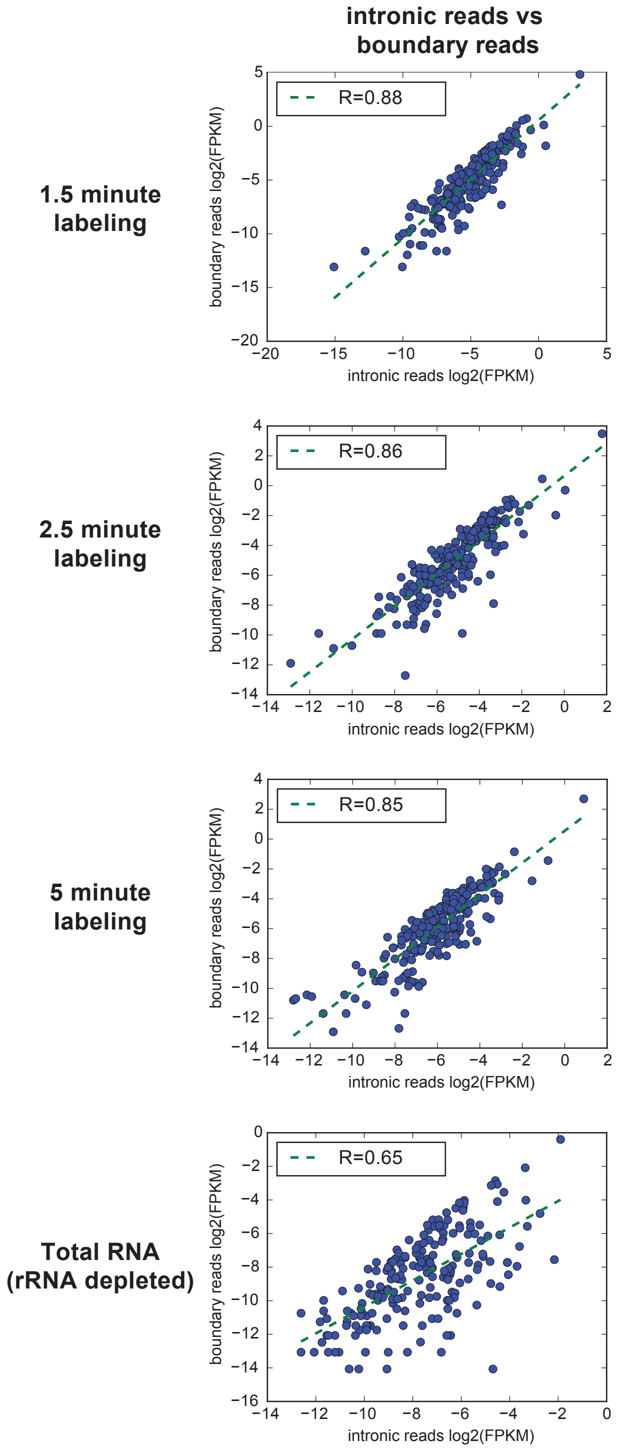

Figure S5. Intron read counts are highly correlated with 5'ss boundary read counts. Intron reads and 5'ss-intron reads were normalized to reads per million (FPKM) and log2 transformed. Shown are the results for the 4tU-labeled samples and rRNA depleted total RNA. Note that for the total RNA sample the boundary read counts were generally much lower, explaining the lower correlation. R indicates Pearson's correlation coefficient.

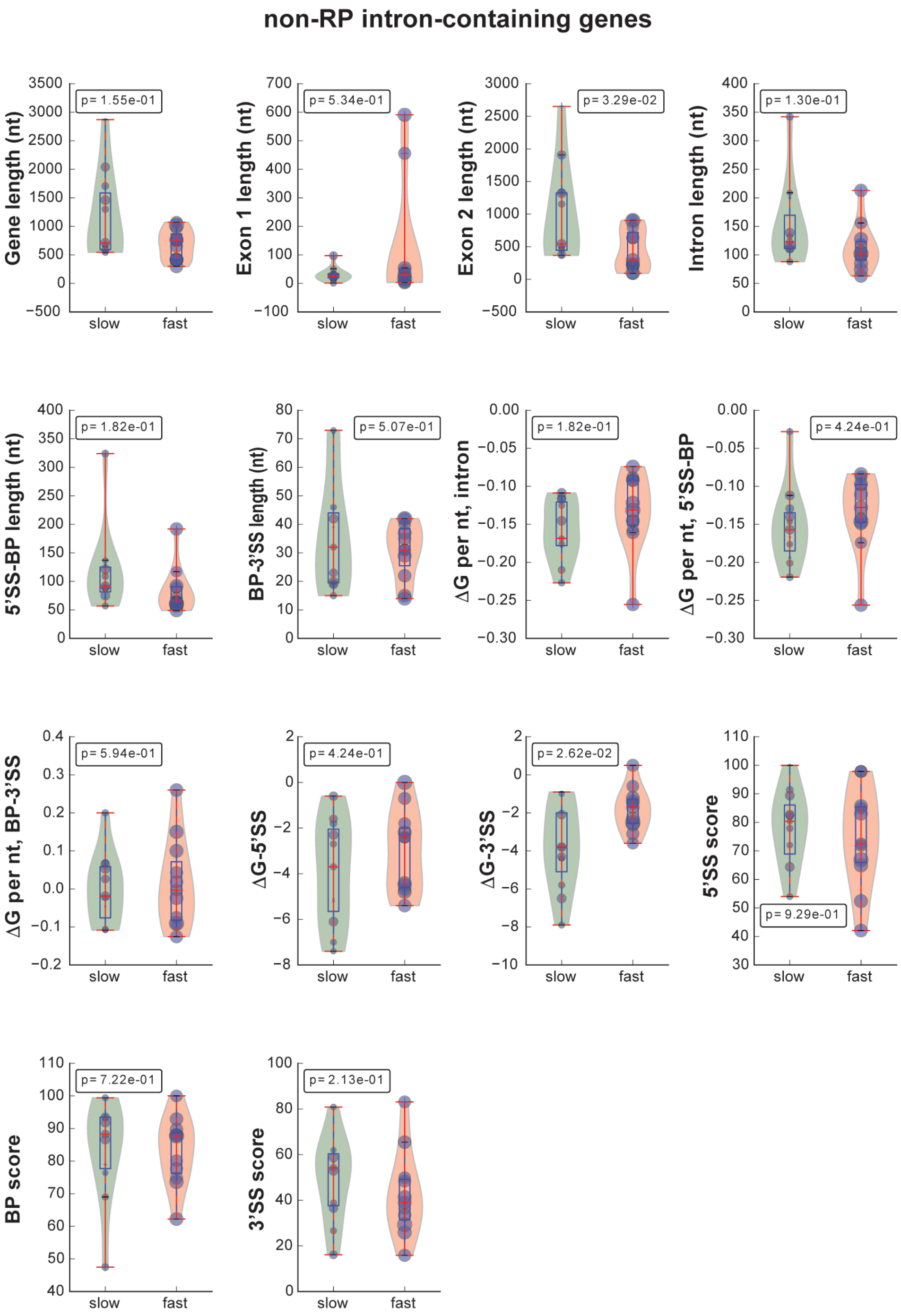

Figure S6. Violin plots of 11 features for the 1/3 fastest and 1/3 slowest non-RP intron-containing genes. The splicing speed is measured by AUC (see Methods). The red horizontal line is the median of the feature, and the red vertical solid line ends at the quartiles of the feature. The dots in the violin box are the samples of each feature, whose sizes are corresponding to its splicing speed.

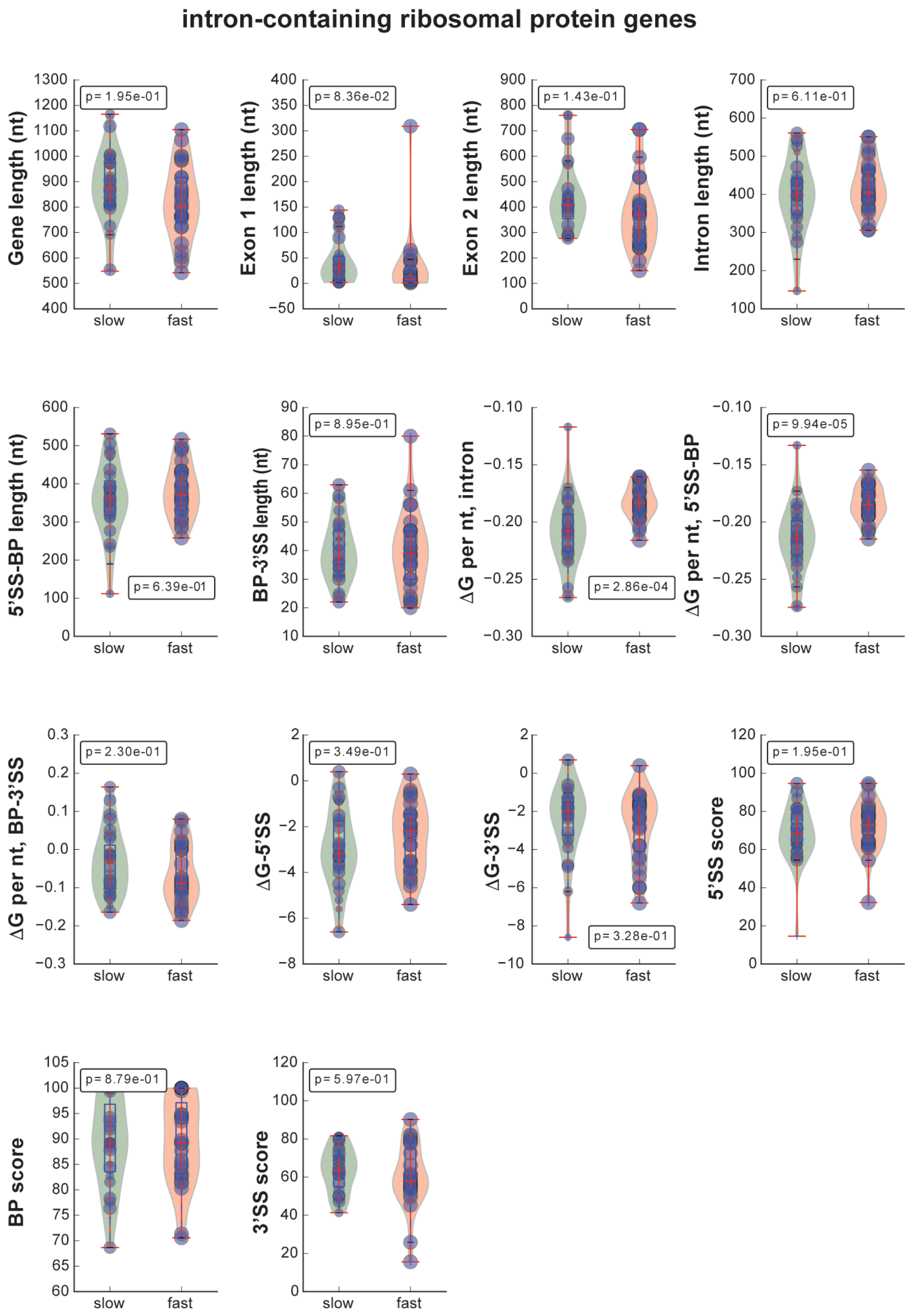

Figure S7. Violin plots of 11 features for the 1/3 fastest and 1/3 slowest RP intron-containing genes. The splicing speed is measured by AUC (see Methods). The red horizontal line is the median of the feature, and the red vertical solid line ends at the quartiles of the feature. The dots in the violin box are the samples of each feature, whose sizes are corresponding to its splicing speed.

Supplementary Methods

This section describes two direct methods and a probabilistic model that we used for estimating splicing ratio from RNA-seq data, and provides a comparison between them by simulation.

Direct methods

The simplest direct method for calculating splicing ratios is to use only reads that are unambiguously assigned to either mature or pre-mRNA. Such reads cover the exon1-exon2 junction for mature mRNA and the intron-exon2 boundary for pre-mRNA. Denoting the number of junction reads as N_m and the number of intron-exon2 boundary reads as N_p the splicing ratio can be estimated as:

$$\varphi = N_m / (N_m + N_p) \quad (1)$$

Tilgner *et al* [1] used a similar measurement to study the subcellular splicing completion with deep RNA-seq data. This direct method has several theoretical advantages: it provides an unbiased estimate of the splicing ratio [2], and does not require any normalization/ bias correction procedure. Nevertheless, it can only be effective for highly covered genes due to the requirement of having a sufficient amount of boundary/ junction reads. Furthermore, very early time points may have extremely few junction reads if splicing times are comparable with the first time point.
In addition to using the junction reads only, the normalized reads counts (RPK, reads per kilo-base pairs) on introns and exons were used in the work of Windhager and colleagues [3]; which could be described with minor modifications as follows,

$$\varphi = 1 - \frac{N_{in} / (l_{in} + l_r)}{N_{ex} / (l_{ex} - l_r + 1)} \quad (2)$$

where l_r is the read length; l_{in} and l_{ex} are the total lengths of intron and exons, respectively; N_{in} and N_{ex} are the corresponding numbers of reads mapped to intron (including partially) or exons. Note that slight modifications may be needed if upstream or downstream exons are short compared to read lengths. This approach does not suffer from the low coverage issues of the junction/ boundary approach, however normalization issues are more problematic, as exon and intron reads will generally be degraded at different rates.

Probabilistic model

In addition to the direct methods, the splicing ratio could be inferred statistically by modelling the total reads as following a mixture distribution, along the lines of the mixture of isoforms (MISO) model for quantifying isoform proportions [2]. The posterior distribution of could be described as follows,

$$
\begin{aligned}
P(\varphi \mid R_{1:N}) &\propto P(\varphi)\times P(R_{1:N}\mid \varphi) \\
&\propto P(\varphi)\times \prod_{n=1}^{N}\sum_{I_n\in\{m,p\}} P(R_{1:N}\mid I_n)P(I_n\mid \varphi)
\end{aligned} \quad (3)
$$

where $R_{1:N}$ is a set of N reads aligned to the given gene; I_n is a latent variable denoting the identity of read R_n belonging to mRNA ($I_n = m$) or pre-mRNA ($I_n = p$). Assuming reads are uniformly distributed given their isoform, we fixed $P(R_{1:N}|I_n)=1/(l_t - l_r + 1)$. The computation of the posterior distribution is broadly along the lines of [2], however, in order to speed up the sampling, we modified the Metropolis-Hasting algorithm by analytically calculating the $P(I_n|\psi)$ given a sampled. Namely the latent variables I_n is collapsed in the Markov chain Monte Carlo (MCMC) sampling. In the following analysis, we use the mean of the posterior distribution of , the mean as the estimated splicing ratio.

Comparison between methods

In order to compare the direct methods and the MCMC sampling method, a simulation was conducted. We set 6 time points with different splicing ratios, for all of the 187 intron-containing genes in yeast. Based on the yeast genome annotated by Ensembl R64-1-1.77, we simulated paired-end reads with length of 100 bp, and average depth of RPK=1000. Then the simulated reads were aligned to the yeast genome.

The comparison results are shown in Figure S3, which illustrates that both direct methods and the MCMC sampler are almost unbiased. However, the direct methods suffer from a greater variability, though method based on intron and exon reads shows smaller variation in latter half time points. The method using intron-exon boundary or exon-exon junction reads is more vulnerable to outliers: several genes have a splicing ratio of zero or one simply because no reads were mapped to junctions or boundaries. This is particularly severe for the later time points. The probabilistic method largely reduced the variation of the estimated splicing ratio compared to any of the direct methods at any time point, especially reducing the extreme cases (i.e., less outliers).

References

1. Tilgner H, Knowles DG, Johnson R, Davis CA, Chakrabortty S, Djebali S, Curado J, Snyder M, Gingeras TR, Guigó R: **Deep sequencing of subcellular RNA fractions shows splicing to be predominantly co-transcriptional in the human genome but inefficient for lncRNAs.** *Genome Res* 2012, **22**:1616–1625.

2. Katz Y, Wang ET, Airoldi EM, Burge CB: **Analysis and design of RNA sequencing experiments for identifying isoform regulation.** *Nat Methods* 2010, **7**:1009–1015.

3. Windhager L, Bonfert T, Burger K, Ruzsics Z, Krebs S, Kaufmann S, Malterer G, L'Hernault A, Schilhabel M, Schreiber S, Rosenstiel P, Zimmer R, Eick D, Friedel CC, Dölken L: **Ultrashort and progressive 4sU-tagging reveals key characteristics of RNA processing at nucleotide resolution.** *Genome Res* 2012, **22**:2031–2042.
